# Demographic and psychological predictors of community pharmacists’ cancer-related conversations with patients: a cross-sectional analysis and survey study

**DOI:** 10.1186/s12913-022-07587-1

**Published:** 2022-02-28

**Authors:** Robert S. Kerrison, Anna Robinson, Hanna Skrobanski, Ghalia Kayal, Aradhna Kaushal, Charlotte Ide-Walters, Adam Todd, Andrew Husband, Shivali Lakhani, Marsha Alter, Christian von Wagner, Lindsay MacDonald

**Affiliations:** 1 School of Health Sciences, University of Surrey, Surrey, UK; 2 School of Pharmacy, Newcastle University, Newcastle, UK; 3 Acaster Lloyd Consulting Ltd, London, UK; 4 Department of Behavioural Science and Health, University College London, London, UK; 5 Cancer Intelligence Team, Cancer Research UK, London, UK; 6 School of Human Sciences, University of Greenwich, London, UK; 7 Middlesex Group of Local Pharmaceutical Committees, London, UK

**Keywords:** Community Pharmacy, Early Diagnosis, Prevention, Screening, Cancer, Bowel Cancer

## Abstract

**Background:**

There is increasing interest in the role of community pharmacy in the early diagnosis and prevention of cancer. This study set out to examine how often community pharmacists (CPs) encourage patients to spot or respond to potential signs and symptoms of cancer, and how often they help people to make an informed decision about taking part in bowel cancer screening.

**Methods:**

Data from 400 UK CPs, who completed the 2018 Cancer Research UK Healthcare Professional Tracker survey, were analysed. The primary outcomes were: ‘how often CPs encourage patients to spot or respond to potential signs and symptoms of cancer’ and ‘how often CPs encourage eligible people to make an informed decision to participate in bowel cancer screening’. Associations between behaviours and demographic and psychological variables (Capability, Opportunity and Motivation) were assessed using multivariate logistic regression.

**Results:**

Most (*n* = 331, 82.8%) CPs reported occasionally, frequently or always encouraging patients to spot or respond to potential signs and symptoms of cancer, while only half (*n* = 203, 50.8%) reported occasionally, frequently or always helping people make an informed decision to participate in bowel cancer screening. Female sex (aOR: 3.20, 95%CI: 1.51, 6.81; *p* < 0.01) and increased Opportunity (aOR: 1.72, 95%CIs: 1.12, 2.64; *p* < 0.05) and Motivation (aOR: 1.76, 95%CIs: 1.37, 2.27; *p* < 0.001) were associated with encouraging patients to spot or respond to potential signs and symptoms of cancer; all three psychological variables were associated with helping people to make an informed decision to participate in bowel cancer screening (Capability: aOR: 1.39, 95%CIs: 1.26, 1.52, *p* < 0.001; Opportunity: aOR: 1.44, 95%CIs: 1.11, 1.87; *p* < 0.01; Motivation: aOR: 1.45, 95%CIs: 1.05, 2.00; *p* < 0.05).

**Conclusions:**

Most CPs encourage patients to spot or respond to potential cancer symptoms, while only half help them make an informed decision to participate in bowel cancer screening. A multifaceted approach, targeting multiple COM-B components, is required to change these behaviours.

**Supplementary Information:**

The online version contains supplementary material available at 10.1186/s12913-022-07587-1.

## Background

Cancer is a leading cause of mortality in the United Kingdom [1]. One and five year survival rates are low compared with countries with comparable healthcare systems (e.g. Australia) [2]. Recognising the signs and symptoms of cancer can play an important part in ensuring early disease diagnosis, which, in turn, can improve survival outcomes for patients [3, 4]. However, most people living in the UK fail to recognise signs and symptoms of cancer, and this has been identified as a major contributor to delayed help-seeking by patients [3, 5, 6].

Screening can also help to detect cancer early, by testing for cancerous or precancerous changes before symptoms develop. However, as with many services, the benefits of screening are limited to those who accept and are eligible to take part in the screening programme. At present, both breast and cervical screening achieve uptake of > 75.0% across the UK population (in 2018, uptake of breast and cervical screening was 81.8% and 76.4%, respectively). By comparison, uptake of bowel cancer screening is lower, with < 60.0% of invitees participating when invited [7].

Increased focus has subsequently been placed on promoting the uptake of bowel cancer screening (also termed ‘colorectal cancer screening’, or ‘CRC screening’), as well as raising awareness of the signs and symptoms of cancer more generally [8]. One healthcare setting that is increasingly helping to deliver these objectives is community pharmacy [9]. Community pharmacy is thought to be particularly well situated to delivering these objectives for two key reasons: first, they are highly accessible to the general population (approximately 90.0% of people in England live within a 20-min walk of a community pharmacy [10]) and, second, they tend to employ people from the local community and are reflective of the local population (hence, people are more comfortable talking to the community pharmacy team about certain conditions) [11]. Community pharmacy, consequently, has the potential to address both cultural and linguistic barriers to information provision, as well as addressing the challenges of healthcare accessibility in deprived areas [10]. The extent to which community pharmacists (CPs) engage with patients in relation to cancer symptoms and bowel cancer screening, however, is unknown.

The aim of the present study was to better understand the working behaviours of CPs in relation to the early diagnosis of cancer. Specifically, this study set out to assess the current practices of CPs in helping patients to: (i) spot and/or respond to potential signs and symptoms of cancer and, (ii) make an informed decision about participating in CRC screening. A secondary aim was to establish the demographic and psychological predictors of these behaviours, to inform the development of future interventions.

## Methods

### Design

Cross-sectional analysis was performed on questionnaire data collected from the 2018 Cancer Research UK Healthcare Professional Tracker Survey (HPTS).

### Data Source

The HPTS is an online survey of UK healthcare professionals, which aims to assess their knowledge, behaviour and beliefs regarding the prevention, early diagnosis and screening of cancer. It was first commissioned in 2010, by a consortium of eight charities (including Cancer Research UK); however, in 2013, Cancer Research UK began commissioning the HPTS independently. The data were collected by Dynata UK’s Healthcare Professional Panel (Dynata, formerly known as ‘Research Now’, are a health market research agency who conduct online sampling and digital data collection) [12]. Consistently surveyed healthcare professionals include Practice Nurses and General Practitioners (GPs), with CPs and Dentists surveyed during intermittent rounds of the survey. Questions cover a variety of topics, including: 1) health professionals’ knowledge of cancer prevention, warning signs, and available screening programmes; 2) health professionals’ practices (*e.g.* discussions with patients) relating to the prevention, early diagnosis and screening of cancer and, 3) Health Professionals’ beliefs (*e.g.* perceived confidence, skills and role) about their contribution to cancer prevention, early diagnosis and screening (Appendix 1).

### Participants

Participants were CPs from across the UK (England, Scotland, Wales and Northern Ireland), who had taken part in the 2018 wave of the HPTS (the most recent wave of the HPTS that included CPs).

### Recruitment

For the 2018 wave, specifically, Pharmacists who self-identify as ‘Community Pharmacists’, were invited by postal invitations (by Dynata), which provided a link to the online survey. The addresses of self-identified CPs were obtained from Wilmington Healthcare: a certified and verified mail list vendor, who have access to > 70.0% of the National Health Service (NHS) workforce [13], and verifies the reported professional status of healthcare professionals by checking their status with the relevant health regulator (e.g. the General Pharmaceutical Council). The vendor state that they update the contact details for health professionals on a regular basis [13].

The HPTS was referred to as a ‘Community Pharmacy’ study in the postal invitation, so that its association with Cancer Research UK was not mentioned, to avoid the survey being suggestive in any way (*i.e.* to reduce bias towards those with greater interest in cancer). Dynata state that approximately 8,000 CPs were sent a postal invitation to take part in the 2018 survey, and that the survey took 10–15 min to complete. CPs were paid £17 (by Dynata) for their participation in the survey.

### Measures

#### Outcome measures

This analysis aimed to understand the extent to which CPs help their patients with issues relating to cancer symptoms and cancer screening. The questionnaire contained two relevant items of interest: 1) encouraging people to spot and/or respond to potential signs and symptoms of cancer (‘In your role as a healthcare professional, how often do you encourage people to spot and/ or respond to potential signs and symptoms of cancer?’) and 2) Helping people make an informed decision about participating in CRC screening (‘In your role as a healthcare professional, how often do you help people make an informed decision about participating in screening for bowel cancer?’). Responses for both outcomes were measured using a five-point Likert scale, with response options: ‘never’, ‘rarely’, ‘occasionally’, ‘frequently’ and ‘always’ (Appendix 1). As certain response options were selected by only a small number of respondents, participant responses have been dichotomised as ‘never/rarely’ and ‘occasionally/frequently/always’ for the purposes of the analysis.

#### Demographic characteristics

Due to the exploratory nature of this research, all demographic variables measured by the survey were selected for use. These included: **sex** (response options: ‘male’, ‘female’), **ethnicity** (response options: ‘White British/Irish/Other’, ‘Asian/Asian British’, ‘Black/Black British’, ‘Chinese or other east and southeast Asian’, ‘Mixed’, ‘Any other mixed’, ‘Other’, ‘Would rather not say’), **geographical region** (response options: ‘Channel Islands’, ‘East of England’, ‘East Midlands’, ‘London’, ‘North East’, ‘North West’, ‘Northern Ireland’, ‘Scotland’, ‘South East’, ‘South West’, ‘Wales’, ‘West Midlands’, ‘Yorkshire’, ‘Not on the map’), **age** (continuous) and **number of years qualified** (continuous).

As certain response options were selected by only a small number of respondents, responses for ‘ethnicity’ were categorised as: ‘**White British/Irish/Other**’ (White British/Irish/Other) and ‘**Any other ethnic group**’ (Asian / Asian British + Black/Black British + Chinese or other east and southeast Asian + Mixed + Any other mixed + Other [no one stated ‘would rather not say’]), while ‘geographic region’ was categorised as ‘**England’** (London, West, East Midlands, Northwest, East, Northeast, Yorkshire, Southeast and Southwest) and ‘**The devolved nations**’ (Scotland, Wales and Northern Ireland [no one stated ‘not on the map’).

#### Psychological variables

Respondents were asked to indicate their agreement (on a five-point Likert scale, ranging from ‘Strongly disagree’ [1], to ‘Strongly agree’ [5]) with seven items relating to how often they encourage people to spot and/or respond to potential signs and symptoms of cancer:

1. It’s part of my role to spot potential signs and symptoms of cancer
2. I feel I have the knowledge to spot potential signs and symptoms of cancer
3. I feel I have the time to discuss and carry out necessary examinations to spot potential signs and symptoms of cancer
4. I feel there is enough training about how to spot potential signs and symptoms of cancer
5. I feel confident in making appropriate referrals for potential signs and symptoms of cancer
6. I believe I can positively influence my patients’ cancer outcomes
7. I feel there is enough training about how to talk to patients/ customers about cancer in general

Similarly, respondents were asked to rate their agreement (again, on a five-point Likert scale with the same response options as above) with six items relating to how often they help people to make an informed decision about taking part in bowel cancer screening:

1. I feel confident helping people make an informed decision about participating in Bowel cancer screening
2. I would like to take a more active role in helping people make an informed decision about participating in Bowel cancer screening
3. I feel I have the knowledge to help people make an informed decision about participating in Bowel cancer screening
4. I feel I have the time to help people make an informed decision about participating in bowel cancer screening
5. I feel I have the skills to help people make an informed decision about participating in bowel cancer screening
6. There is enough training available about how I can counsel people about the benefits and risks of participating in bowel cancer screening

#### Mapping psychological items onto the COM-B (Capability, Opportunity, Motivation and Behaviour) model

**Fig. 1:**
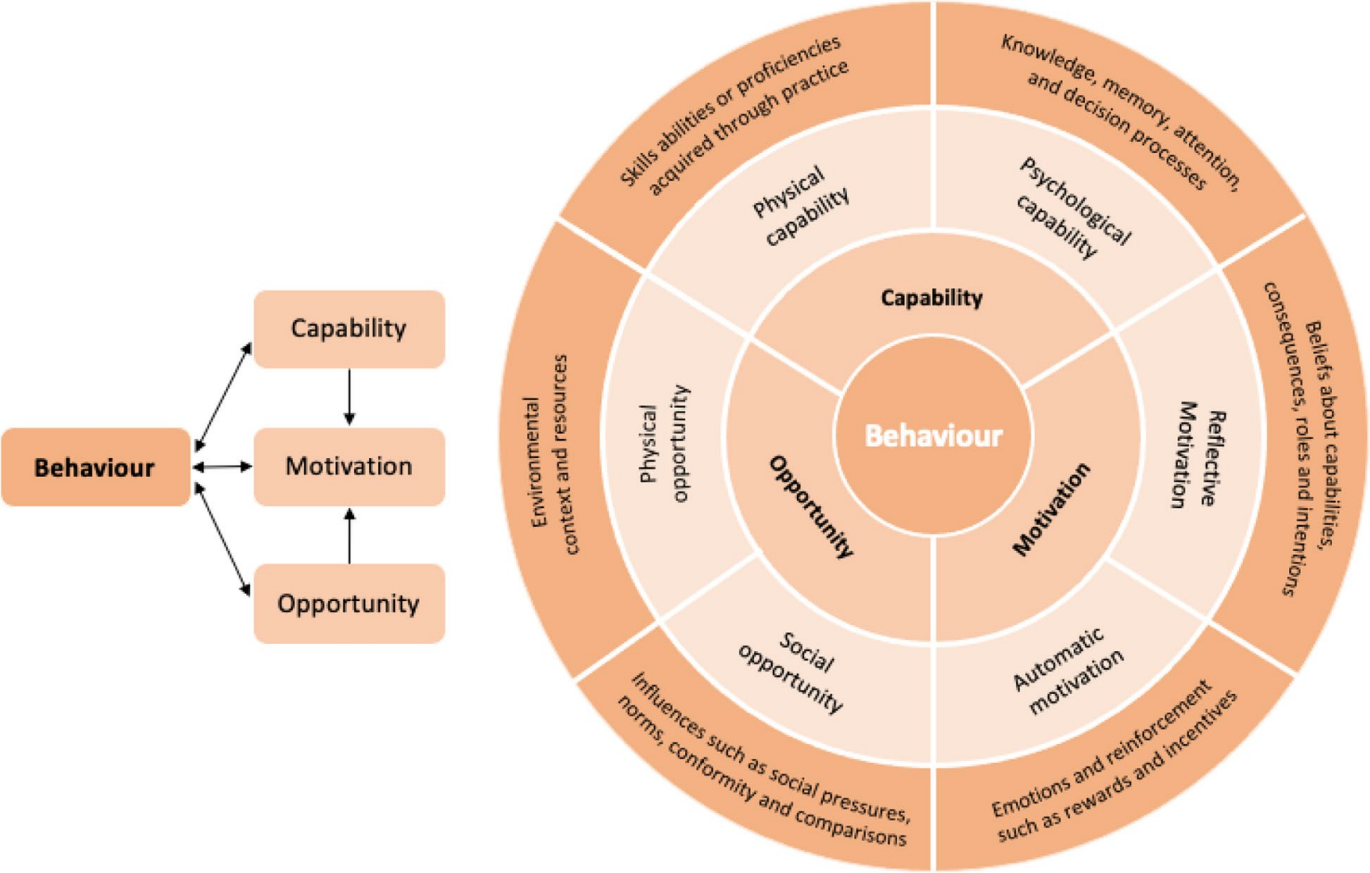
The COM-B model, as adapted from Michie et al. [14, 15] and McDonagh et al. [17]

In order to assess the current practices of CPs and aid interpretation of the data, psychological variables were mapped onto the Capability, Opportunity, Motivation, Behaviour (COM-B) model by Michie et al.(Table 1 and 2) [14, 15]. The COM-B model stipulates that behaviours are the result of three-way interactions between capability (both physical and psychological), opportunity (both social and physical) and motivation (both reflective and automatic) components (as demonstrated in Fig. 1). The COM-B model lies at the centre of the behaviour change wheel, a framework for developing behavioural interventions, and can be used as a starting point for intervention development [16]. By mapping psychological items to the COM-B model, we hope to identify areas of focus to best support CP behaviour change using a theory-based approach.

**Table 1:** List of the psychosocial variables relating to supporting CP users with signs and symptoms of cancer and the TDF domains and COM-B components which they mapped on to.

**Capability**
‘I feel there is enough training about how to talk to patients/ customers about cancer in general’
‘I feel there is enough training about how to spot potential signs and symptoms of cancer’
‘I feel confident in making appropriate referrals for potential signs and symptoms of cancer’
‘I feel I have the knowledge to spot potential signs and symptoms of cancer’
**Opportunity**
‘I feel I have the time to discuss and carry out necessary examinations to spot potential signs and symptoms of cancer
**Motivation**
‘It’s part of my role to spot potential signs and symptoms of cancer’
‘I believe I can positively influence my patients’ cancer outcomes’

**Table 2:** List of the psychosocial variables relating to supporting CP users making informed choice about CRC screening and the TDF domains and COM-B components which they mapped on to.

**Capability**
‘I feel I have the skills to help people make an informed decision about participating in bowel cancer screening’
‘I feel I have the knowledge to help people make an informed decision about participating in Bowel cancer screening’
‘I feel confident helping people make an informed decision about participating in Bowel cancer screening’
‘There is enough training available about how I can counsel people about the benefits and risks of participating in bowel cancer screening’
**Opportunity**
‘I feel I have the time to help people make an informed decision about participating in bowel cancer screening’
**Motivation**
‘I would like to take a more active role in helping people make an informed decision about participating in Bowel cancer screening’

The process of mapping psychological variables onto the COM-B model was achieved through detailed discussion between the authors, using the definitions for Capability, Opportunity and Motivation as described by Michie et al. [14, 15]. Items were first coded by CvW, whose coding was reviewed and agreed by RK and HS. Factor analysis with principal component factor analysis and Oblimin rotation was then performed to test whether there was a high correlation between the variables as grouped by the researchers (the loading criterion was set at 0.40 and above) [18]. The results confirmed high correlation between the items / within the ascribed categories (Appendix 2); scales were subsequently computed for each of the COM-B components by calculating the sum of their scores (values were assigned to the following responses: Strongly disagree = 1; Disagree = 2; Neither agree nor disagree = 3; Agree = 4; Strongly agree = 5). For the ‘encourage people to spot and/or respond to potential signs and symptoms of cancer’ outcome-specific variables, this led to the creation of a multi-item Capability scale ranging from 4–20, a single-item Opportunity scale ranging from 1–5, and a two-item Motivation scale ranging from 2–10 (Table 1). For the ‘help people to make an informed decision about participating in screening for bowel cancer’ outcome-specific variables, the process led to the creation of a multi-item Capability scale ranging from 4–20, a single-item Opportunity scale ranging from 1–5, and a single-item Motivation scale ranging from 1–5 (Table 2).

### Missing data

As the response format for all survey items was forced choice (*i.e.* respondents could not progress through the questionnaire until a response option for each question had been selected), there were no missing data for any of the variables.

### Quality control

Prior to analysis by the research team, the following checks were carried out by Dynata:

1. checking of the completeness/appropriateness of open-ended responses [19]: data was reviewed to ensure that open-ended responses were logical (*e.g.* whether respondent’s ‘number of years qualified’ was accurate in regard to their age), and there were no vague or incomplete answers;
2. identification of ‘speeders’ [16]: data from respondents who spent less than a third of the median length of survey time were removed. This ensured the removal of respondents who, it was felt by Dynata, had completed the survey too quickly to have read or understood the survey items efficiently.

These quality checks were carried out to ensure the removal of any respondents who appeared to be rushing, or not paying sufficient attention to the survey items; an approach previously described by Conrad et al., (2017). Said checks resulted in the removal of seven respondents.

### Patient and public involvement

There was no patient and public involvement in this study.

### Statistical analyses

Descriptive statistics were used to describe the demographic characteristics of the sample. Binary logistic regression was used to test for multivariate associations between co-variates (both demographic and psychological) and how often community pharmacists (i) encourage patients to spot or respond to potential signs and symptoms of cancer and, (ii) help patients to make an informed decision about participating in CRC screening. Ordinal regression was also performed to test for these associations, to test whether associations were present, before dichotomising the data. The amount of variance explained by each model was assessed using the Nagelkerke R^2^ statistic. All analyses were performed using SPSS (version 25).

## Results

### Factor analysis of COM-B items for encouraging patients to spot and respond to potential warning signs of cancer

After Oblimin rotation, a total of 7 items loaded significantly onto 3 factors (labelled ‘Capability’, ‘Opportunity’ and ‘Motivation’) for ‘Encouraging patients to spot and respond to potential warning signs of cancer’. Collectively, the three factors explained a total of 79.72% of the variance.

The results of the analysis are shown in Appendix 2A. The Capability items were loaded together (these four items loaded onto the first factor and ranged from 0.67 to 0.90) and accounted for 59.45% of the variance. In addition, the Motivation items loaded together (these two items loaded onto the third factor and ranged from 0.90 and 0.71) and accounted for a further 12.45% of the variance. Finally, the one item for Opportunity loaded separately (with a loading value of 0.93), and accounted for an additional 7.76% of the variance. Zero items were deleted because of a loading lower than 0.40.

### Factor analysis of COM-B items for encouraging patients to spot and respond to potential warning signs of cancer

After Oblimin rotation, a total of 6 items loaded significantly onto 3 factors (labelled ‘Capability’, ‘Opportunity’ and ‘Motivation’) for ‘Helping people make an informed decision about bowel cancer screening’. Collectively, the three factors explained a total of 82.41% of the variance.

The results of the analysis are shown in Appendix 2B. The Capability items were loaded together (these four items loaded onto the first factor and ranged from 0.79 to 0.89) and accounted for 54.79% of the variance. The Motivation and Opportunity items loaded separately (with loading values of 0.99 and 0.98, respectively) and accounted for a further 16.82% and 10.80% of the variance, respectively. Zero items were deleted because of a loading lower than 0.40.

### Sample characteristics

In total, 400 CPs completed the questionnaire. Respondents were predominantly male (*n* = 291, 67.8%), of White British/Irish/Other ethnicity (*n* = 208, 52.0%) and based at a community pharmacy in England (*n* = 337, 84.2%). The mean age of participants was 43 years (SD: 10.0), and the mean number of years qualified was 19.4 (SD: 10.6) (Table 3). The mean Capability, Opportunity and Motivation scores for encouraging people to spot and / or respond to potential signs and symptoms of cancer were 11.6 (SD: 3.4), 2.64 (SD: 1.1) and 7.2 (SD: 1.6), respectively. The mean Capability, Opportunity and Motivation scores for helping people make an informed decision about taking part in bowel cancer screening were 11.9 (SD: 3.5), 2.8 (SD: 1.1) and 3.7 (SD: 0.9), respectively.

**Table 3:** Sample characteristics

	*n* (%)
**Sex**	
Male	271 (67.8)
Female	129 (32.2)
**Ethnicity**	
White British/Irish/Other	208 (52.0)
Any other ethnic group	43 (48.0)
**Region**	
England	337 (84.2)
Devolved nations of the UK: Northern Ireland, Scotland, Wales	63 (15.8)
**Number of years qualified, Mean (SD)**	
Number of years (Continuous)	19.84 (10.6)
**Age, Mean (SD)**	
Number of years (Continuous)	43.30 (10.0)

## Analysis

### Encourage people to spot and/ or respond to potential signs and symptoms of cancer

**Fig. 2:**
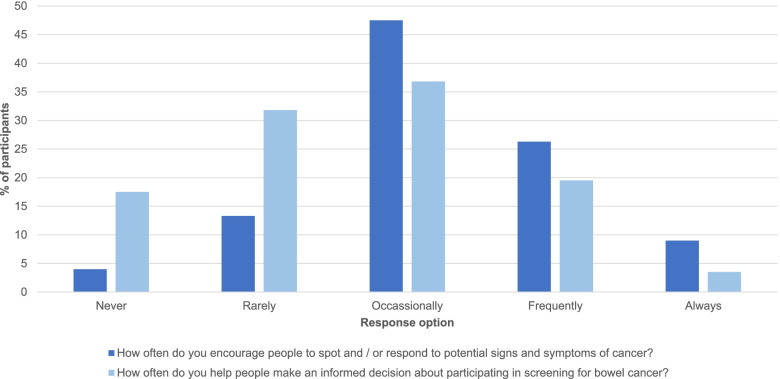
Distribution of responses to 'encouraging people to spot cancer symptoms' and 'helping people make an informed decision about participating in bowel cancer screening'

Table 4 shows the percentage of CPs who reported helping patients to spot and/or respond to potential signs and symptoms of cancer. The majority (*n* = 331, 82.8%) reported encouraging patients occasionally, frequently, or always; while only 1 in 5 (*n* = 69, 17.3%) reported never or rarely encouraging patients (Table 4). The distribution of the responses, prior to being dichotomised, is demonstrated in Fig. 2.

**Table 4:** Demographic and psychological predictors of encouraging people to spot and/ or respond to potential signs and symptoms of cancer

	**Never/Rarely** **(*n* = 69)**	**Occasionally/Frequently/Always** **(*n* = 331)**	**Adjusted OR** **(95%CI)**
**Demographic factors**			
**Sex *n* (%)**			
Male	55 (20.3)	216 (79.7)	1.00
Female	14 (10.9)	115 (89.1)	**3.20** (1.51, 6.81)**
**Ethnicity, *n* (%)**			
White British/Irish/Other	27 (13.0)	181 (87.0)	1.00
Other + Black + Chinese	42 (21.9)	150 (78.1)	0.80 (0.40, 1.58)
**Region, *n* (%)**			
England	59 (17.5)	278 (82.5)	1.00
Devolved nations of the UK: Northern Ireland, Scotland, Wales	10 (15.9)	53 (84.1)	1.10 (0.43, 2.82)
**Age, Mean (SD)**			
Years (Continuous)	42.2 (9.9)	43.5 (10.0)	0.96 (0.86, 1.06)
**Number of years qualified, Mean (SD)**			
Years (Continuous)	18.2 (10.1)	20.2 (10.7)	1.08 (0.98, 1.20)
**COM-B factors, Mean (SD)**			
Capability (4 – 20)	9.2 (3.2)	12.1 (3.2)	1.06 (0.92, 1.21)
Opportunity (1 – 5)	2.0 (0.9)	2.8 (1.4)	1.72 (1.12, 2.64)*
Motivation (2 – 10)	5.7 (1.7)	2.6 (1.1)	1.76 (1.37, 2.27)***
***R2* = 0.34**			

^*^P < 0.05

^**^P < 0.05

^***^ *P* < 0.01

The logistic regression explained 34.0% of the variance (*R*^*2*^ = 0.34;Table 4). Female sex was the only demographic factor associated with being more likely to occasionally, frequently or always encourage patients (89.1% of female CPs responded ‘occasionally, frequently or always’, compared with 79.1% of male CPs: adjusted odds ratio [aOR]: 3.20, 95%CI: 1.51, 6.81; *p* < 0.01). Of the COM-B variables, increased Opportunity and increased Motivation were both associated with occasionally, frequently or always encouraging patients to spot and/or respond to potential signs and symptoms of cancer (the mean Opportunity scores were 2.80 for those who responded ‘occasionally, frequently always’ and 2.00 for those who responded ‘never/rarely’: aOR: 1.72, 95%CIs: 1.12, 2.64; *p* < 0.05; the mean Motivation scores were 5.7 and 2.6 for those who responded ‘occasionally, frequently always’ and ‘never/rarely’, respectively: aOR: 1.76, 95%CIs: 1.37, 2.27; *p* < 0.001). The results were the same for the ordinal regression model, which confirmed that female sex, increased Opportunity and increased Motivation were all predictors of encouraging patients to spot or respond to potential signs and symptoms of cancer (in the ordinal regression, 35.0% of the variance was explained: *R*^*2*^ = 0.35; Appendix 4a).

### Help people make an informed decision about participating in screening for bowel cancer

Table 5 shows the percentage of CPs who reported helping people make an informed decision about participating in screening for bowel cancer. Just over half (*n* = 203, 50.8%) reported encouraging patients occasionally, frequently, or always, while slightly less than half (*n* = 197, 49.2%) reported never or rarely encouraging patients (Table 5). The distribution of the responses, prior to being dichotomised, is demonstrated in Fig. 2.

**Table 5:** Demographic and psychological predictors of helping people make an informed decision about participating in screening for Bowel Cancer?

	**Never/Rarely** **(*n* = 197)**	**Occasionally/Frequently/Always** **(*n* = 203)**	**Adjusted OR** **(95%CI)**
**Demographic factors**			
**Sex *n* (%)**			
Male	137 (50.6	134 (49.4)	1.00
Female	60 (46.5)	69 (53.5)	1.39 (0.82, 2.35)
**Ethnicity, *n* (%)**			
White British/Irish/Other	98 (47.1)	110 (52.9)	1.00
Any other ethnicity	99 (51.6)	93 (48.4)	0.90 (0.53, 1.53)
**Region, *n* (%)**			
England	167 (49.6)	170 (50.4)	1.00
Devolved nations of the UK: Northern Ireland, Scotland, Wales	30 (47.6)	33 (52.4)	1.19 (0.59, 2.44)
**Age, Mean (SD)**			
Years (Continuous)	42.2 (9.5)	44.4 (10.5)	0.95 (0.86, 1.04)
**Number of years qualified, Mean (SD)**			
Years (Continuous)	18.1 (10.1)	21.1 (10.9)	1.07 (0.98, 1.17)
**COM-B factors, Mean (SD)**			
Capability (4 – 20)	10.0 (3.2)	**13.7 (2.8)**	1.39 (1.26, 1.52)***
Opportunity (1 – 5)	2.4 (1.0)	**3.2 (1.0)**	1.44 (1.11, 1.87)**
Motivation (1 – 5)	3.4 (1.0)	**4.1 (0.8)**	1.45 (1.05, 2.00)*
***R2* = 0.41**			

^*^P < 0.05

^**^P < 0.05

^***^P < 0.01

The logistic regression explained 41.0% of the variance (*R*^*2*^ = 0.41;Table 5;). There were no demographic differences between CPs who never/rarely or occasionally/frequently always help people make an informed decision about participating in screening for bowel cancer. All three COM-B variables were associated with helping people make an informed decision about participating in bowel cancer screening (the mean Capability scores were 13.7 and 10.0 for those who responded ‘occasionally, frequently always’ and ‘never/rarely’, respectively: aOR: 1.39, 95%CIs: 1.26, 1.52, *p* < 0.001; the mean Opportunity scores were 3.2 and 2.4 for those who responded ‘occasionally, frequently always’ and ‘never/rarely’, respectively: aOR: 1.44, 95%CIs: 1.11, 1.87; *p* < 0.01; the mean Motivation scores were 3.1 and 3.4 for those who responded ‘occasionally, frequently always’ and ‘never/rarely’, respectively: aOR: 1.45, 95%CIs: 1.05, 2.00; *p* < 0.05;Table 5;). The results were the same for the ordinal regression model, which confirmed increased Capability, increased Opportunity and increased Motivation were all predictors of helping people make a decision about participating in screening for bowel cancer occasionally, frequently or always (in the ordinal regression, 35.0% of the variance was explained: *R*^*2*^ = 0.41; Appendix 4b).

## Discussion

### Statement of principal findings

This study found that most CPs encourage patients to spot and respond to potential signs and symptoms of cancer, while less than half help people to make an informed decision to take part in bowel cancer screening. This study also found that all three components of the COM-B model (i.e. Capability, Opportunity and Motivation) are linked to helping patients make an informed decision about taking part in bowel cancer screening, while only Opportunity and Motivation are linked with encouraging patients to spot and / or respond to potential signs and symptoms of cancer. Importantly, our study identified specific demographic groups who are more frequently engaged with these behaviours, meaning that we are able to make recommendations about not only the psychological variables that interventions seeking to change behaviour should target, but the populations they could be targeted towards as well. Finally, our study highlighted that, while the demographic and psychological variables examined account for a large amount of the variation in CPs behaviour (i.e. 34–41%), it does not account for all of the variance. This indicates that there are other important factors that need to be considered and explored in future research.

### The meaning of the study: possible explanations and implications for clinicians and policymakers

Previous studies have suggested that further training may increase the confidence of CPs in helping patients to recognise signs and symptoms of cancer [20–22], but have lacked detail on how to achieve this. The present study suggests that a broad approach, targeting both Motivation and Opportunity, specifically, is required. Furthermore, the findings of this study highlight the specific aspects of these components that could be targeted to change behaviour. For example, as shown in Table 1, targeting a CP’s perception of their professional role and identity would be one possible target for intervention development in relation to improving Motivation. The next logical step for policy makers would be to map these psychological targets onto the Behaviour Change Wheel / Behaviour Change Technique Taxonomy, to identify behaviour change techniques that are likely to be effective at modifying these psychological targets and, therefore, CP behaviour [15].

Previous research has also suggested that conversations about cancer screening are a less familiar behaviour to CPs, compared to educating patients about symptom recognition, and one which is usually initiated by the patient [23]. For example, a previous survey study found that 15% of CPs stated that their confidence to discuss bowel cancer screening was ‘poor’ or ‘very poor’, and that only half were aware that bowel cancer screening was for people who did not have symptoms [20]. The present research builds on these findings, by highlighting that it is not just Capability which is important (both of which come under the ‘Capability construct of COM-B), but factors related to Opportunity and Motivation as well (as well as several other factors related to Capability). The need to target all three components of the COM-B model suggests that a more holistic and rounded approach is required to facilitate behaviour change in this context. Indeed, if screening is to become part of a regular conversation initiated by CPs, it may need to be embedded within a pathway, or made part of public health promotional campaigns. One possible strategy would be to inform CPs of patient’s screening outcomes; another would be to streamline processes to enable CPs to request a replacement screening kit for their patients, or for them to be supplied with leaflets by the NHS, which can then be used to facilitate conversations with patients (as well as taken away by patients to provide information, aid memory and facilitate behaviour change).

### Strengths and weaknesses of the study

This study had several strengths. First, it contained a diverse range of CPs from different ethnic backgrounds and levels of experience, making the findings more generalisable to the UK CP population (National data suggest 44.8% of CPs are of White British/Irish/Other ethnicity) [17]. Second, it used a forced choice design, meaning that there were no missing data for any of the participants. Third, several quality control measures were implemented, including checking of open-ended responses for completeness (e.g. age, number of years qualified), as well as the time spent on each question, with several cases consequently removed (e.g. speeders). Fourth, the study was grounded in theory, making use of the COM-B Model, allowing for the development of theory-based interventions via the Behaviour Change Wheel. Fifth, the study included a wide range of demographic and psychological variables, and explained a large amount of the variance in CPs’ cancer-related behaviours. Finally, the study used factor analysis to test the validity of the COM-B components, and found high construct validity for the three components, allowing greater confidence to be placed in the trustworthiness of the results.

This study also had several limitations. First, it used self-reported measures for behaviour and may, therefore, be subject to self-report bias, with CPs potentially self-reporting higher frequencies of desired behaviours than practiced (e.g. due to expectations that Pharmacists provide additional healthy living services, as per their contractual framework). Second, this study used a self-selected sample (only 5% of invited CPs [400 of 8,000] completed the questionnaire), and may, in addition, be subject to self-selection bias, with participating CPs being more engaged with health promotion activities than the general population of CPs (*(although it is important to note that CPs were not informed the questionnaire was related to cancer, or health promotion [just health] and the demographics of our sample broadly reflect those of the wider CP population in terms of age, sex and ethnicity [the mean age of our sample was 43 years, while that of the wider CP population is 41; 68% of our sample were male, while 62% of the wider population are male; 52% of our sample identified as being of a White ethnic background, while 46% of the wider population identify as being of a White ethnic background]) *[17]) [24]. Third this study used cross-sectional data, as opposed to longitudinal data and, as such, it was not possible to assess how CP cancer-related behaviours have changed over time (it is possible, for example, that during flu season, there is less time for CPs to engage in cancer-related discussions with patients). Fourth, only a small number of CPs from the devolved nations and some ethnic groups participated in the study. As such, there was low power to assess regional and ethnic group differences in behaviour. Fifth, as data on the individual pharmacy CPs were registered with were not collected, it is not possible to say whether the 400 CPs participating in this study were registered with 400 different pharmacies. This may further reduce the representativeness of the sample and the generalisability of it’s findings. Finally, the survey lacked more quantifiable data on how many patients report to their CPs with cancer-related queries, which would help enable more accurate estimates about the potential benefit and cost effectiveness of training CPs.

### Unanswered questions and future research

This study highlights several areas for future research. First, future research is needed to understand why female CPs are more likely to encourage patients to spot and respond to potential signs and symptoms of cancer than male CPs. Second, further research is needed to identify additional factors that affect whether CPs engage in these behaviours (the variables included in our study only accounted for 34–41% of the variance in CPs behaviour). It may be, for example, that community pharmacies that see smaller numbers of patients on a daily basis are able to engage in cancer-related conversations more frequently. Third, additional data on the patient-reported experience of CP-led cancer-related consultations is needed to determine whether there are areas for improvement. Finally, future research is needed to explore the role and behaviours of other community pharmacy staff, such as Healthcare Assistants, to understand what role they may play in initiating cancer screening-related conversations with patients in the community pharmacy setting.

## Conclusions

This study demonstrates that most CPs encourage patients to recognise and respond to potential signs and symptoms of cancer, but that less than half help people to make an informed decision to take part in bowel cancer screening. As such, the results of this study highlight an emerging gap where CPs could play a larger role in helping people to make an informed decision about taking part in bowel cancer screening (one which might be even greater than this study suggests, given the likelihood of self-selection bias by CPs who are more interested in engaging with health promotion activities). The findings from this work suggest that holistic approaches to changing CP behaviours are required to achieve behaviour change, with multiple components of the COM-B model of behaviour being significant predictors of cancer-related working behaviours.

## Supplementary Information

**Additional file 1. **

**Additional file 2. **

**Additional file 3. **

**Additional file 4. **

## Data Availability

The data used in this study are available from Open Science Framework: https://osf.io/qutwr/

## Abbreviations

aOR: Adjusted Odds Ratio
CI: Confidence Interval
CP: Community Pharmacist
CRC: Colorectal Cancer
GP: General Practitioner
HPTS: Healthcare Professional Tracker Survey
NHS: National Health Service
SD: Standard Deviation
