# Correction to: Demographic and psychological predictors of community pharmacists’ cancer-related conversations with patients: a cross-sectional analysis and survey study

**DOI:** 10.1186/s12913-022-07746-4

**Published:** 2022-03-15

**Authors:** Robert S. Kerrison, Anna Robinson, Hanna Skrobanski, Ghalia Kayal, Aradhna Kaushal, Charlotte Ide-Walters, Adam Todd, Andrew Husband, Shivali Lakhani, Marsha Alter, Christian von Wagner, Lindsay MacDonald

**Affiliations:** 1 School of Health Sciences, University of Surrey, Surrey, UK; 2 School of Pharmacy, Newcastle University, Newcastle, UK; 3 Acaster Lloyd Consulting Ltd, London, UK; 4 Department of Behavioural Science and Health, University College London, London, UK; 5 Cancer Intelligence Team, Cancer Research UK, London, UK; 6 School of Human Sciences, University of Greenwich, London, UK; 7 Middlesex Group of Local Pharmaceutical Committees, London, UK

**Correction to: BMC Health Serv Res 22, 268 (2022)**

**https://doi.org/10.1186/s12913-022-07587-1**

Following publication of the original article [1], the authors identified an error in the author affiliations’ list.

The author affiliations have been updated in this correction article and the original article [1] has been corrected.
