# Supplementary material for: Demographic and psychological predictors of community pharmacists’ cancer-related conversations with patients: a cross-sectional analysis and survey study
Source: BMC Health Serv Res. 2022 Feb 28;22:268. doi: 10.1186/s12913-022-07587-1 (PMC8883634; doi:10.1186/s12913-022-07587-1)
Supplement: Supplementary file 1 — Additional file 1. [file 12913_2022_7587_MOESM1_ESM.docx]

**Healthcare Professionals Tracker 2018**

QGEN. Are you….

1. Male
2. Female

QAGE. How old are you?

QREG. Which region do you live in?

1. Channel Islands
2. East of England
3. East Midlands
4. London
5. North East
6. North West
7. Northern Ireland
8. Scotland
9. South East
10. South West
11. Wales
12. West Midlands
13. Yorkshire/ Humberside
14. Not on Map **[CLOSE]**

QEt. To which ethnic group do you consider you belong?

1. White (British/Irish/Other)
2. Black/Black British (Caribbean/African/Other)
3. Asian/Asian British (Indian/Pakistani/Bangladeshi/Other Asian)
4. Chinese or other east & south east Asian
5. Mixed (White and Black Caribbean/White and Black African/White and Asian)
6. Any other mixed
7. Other
8. Would rather not say

S2. What year did you qualify as Pharmacist?

**PART FOUR: EARLY DIAGNOSIS**

*The next set of questions are about the early diagnosis of cancer*

In your role as a healthcare professional, how often do you do the following?

|  | 1. Never | 2. Rarely | 3. Occasionally | 4. Frequently | 5. Always |
| --- | --- | --- | --- | --- | --- |
| 1. Encourage people to spot and/ or respond to potential signs and symptoms of cancer |  |  |  |  |  |

QUESTION 13: **2015**

In your role as a healthcare professional, please say how much you agree or disagree with the following statements.

|  | 1. Strongly disagree | 2. Disagree | 3. Neither agree nor disagree | 4. Agree | 5. Strongly agree |
| --- | --- | --- | --- | --- | --- |
| 10. It’s part of my role to spot potential signs and symptoms of cancer |  |  |  |  |  |
| 14. I feel I have the knowledge to spot potential signs and symptoms of cancer |  |  |  |  |  |
| 15. I feel I have the time to discuss and carry out necessary examinations to spot potential signs and symptoms of cancer |  |  |  |  |  |
| 11. I feel there is enough training about how to potential signs and symptoms of cancer. |  |  |  |  |  |
| 9.I feel confident in making appropriate referrals for potential signs and symptoms of cancer |  |  |  |  |  |
| 12. I believe I can positively influence my patients’ cancer outcomes |  |  |  |  |  |
| 13. I feel there is enough training about how to talk to patients/ customers about cancer in general. |  |  |  |  |  |

**PART FIVE: SCREENING**

*The next set of questions are about NHS national cancer screening programmes*

QUESTION 16: **PROCAM 2015**

Is there currently a NHS national cancer screening programme for bowel cancer?

1. Yes
2. No
3. Not sure

QUESTION 17: **2015**

Which, if any, of the following are currently ~~routinely~~ used in the bowel cancer screening programme as a first line test? *Please tick all that apply.*

1. Faecal occult blood (FOB)
2. Bowel scope/ Flexible sigmoidoscopy
3. CT colonography
4. Computed tomography
5. Digital Rectal Exam
6. Barium enema
7. Faecal immunochemical testing (FIT)
8. Don’t know

QUESTION 19A:

At what ages are adults in your region sent an invitation to attend for a Bowelscope/ Flexible sigmoidoscopy test?

97. Unavailable in my region

QUESTION 19B:

Do you currently promote uptake to the following bowel screening tests with your patients/ customers?

|  | 1. Yes | 2. No | 3. Unavailable in my region |
| --- | --- | --- | --- |
| 1. Faecal occult blood (FOB)~~/~~ |  |  |  |
| 2. Faecal immunochemical testing (FIT) |  |  |  |
| 3. Bowelscope/ Flexible sigmoidoscopy |  |  |  |

QUESTION 19C:

Please tell us the reasons why you do not currently promote uptake to Faecal occult blood (FOB) screening?

QUESTION 19D:

Please tell us the reasons why you do not currently promote uptake to Faecal immunochemical testing (FIT) screening?

QUESTION 19E:

Please tell us the reasons why you do not currently promote uptake to Bowelscope/ Flexible sigmoidoscopy screening?

QUESTION 19F:

How aware are you of the different uses of Faecal immunochemical testing (FIT) in screening and symptomatic pathways?

|  | 1. Very aware | 2. Somewhat aware | 3. Unaware |
| --- | --- | --- | --- |
| 1. FIT for screening |  |  |  |
| 2. FIT for symptomatic patients |  |  |  |

QUESTION 26: **2015**

In your role as a healthcare professional, how often do you help people make an informed decision about participating in screening for…?

|  | 1. Never | 2. Rarely | 3. Occasionally | 4. Frequently | 5. Always |
| --- | --- | --- | --- | --- | --- |
| Bowel cancer |  |  |  |  |  |

QUESTION 27: **2015**

Thinking about your role as a healthcare professional, please say how much you agree or disagree with the following statements.

|  | 1. Strongly disagree | 2. Disagree | 3. Neither agree nor disagree | 4. Agree | 5. Strongly agree |
| --- | --- | --- | --- | --- | --- |
| **It’s part of my role to help people make an informed decision about participating in Bowel cancer screening** |  |  |  |  |  |
| **I feel confident helping people make an informed decision about participating in** **Bowel cancer screening** | 1. Strongly disagree | 2. Disagree | 3. Neither agree nor disagree | 4. Agree | 5. Strongly agree |
| **I would like to take a more active role in helping people make an informed decision about participating in Bowel cancer screening** | 1. Strongly disagree | 2. Disagree | 3. Neither agree nor disagree | 4. Agree | 5. Strongly agree |
| **I feel I have the knowledge to help people make an informed decision about participating in Bowel cancer screening** | 1. Strongly disagree | 2. Disagree | 3. Neither agree nor disagree | 4. Agree | 5. Strongly agree |

|  | 1. Strongly disagree | 2. Disagree | 3. Neither agree nor disagree | 4. Agree | 5. Strongly agree |
| --- | --- | --- | --- | --- | --- |
| 1. I feel I have the time to help people make an informed decision about participating in bowel cancer screening |  |  |  |  |  |
| 1. I feel I have the skills to help people make an informed decision about participating in bowel cancer screening |  |  |  |  |  |
| 1. There is enough training available about how I can counsel people about the benefits and risks of participating in bowel cancer screening |  |  |  |  |  |
