# Supplementary material for: Demographic and psychological predictors of community pharmacists’ cancer-related conversations with patients: a cross-sectional analysis and survey study
Source: BMC Health Serv Res. 2022 Feb 28;22:268. doi: 10.1186/s12913-022-07587-1 (PMC8883634; doi:10.1186/s12913-022-07587-1)
Supplement: Supplementary file 2 — Additional file 2. [file 12913_2022_7587_MOESM2_ESM.docx]

**Appendix 2A.** Factor analysis output for ‘Encouraging patients to spot and respond to potential signs and symptoms of cancer’

| **Structure Matrix** | | | |
| --- | --- | --- | --- |
|  | Component | | |
|  | 1 | 2 | 3 |
| I feel confident in making appropriate referrals for potential signs and symptoms of cancer | .745 | .692 | .370 |
| It’s part of my role to spot potential signs and symptoms of cancer | .297 | .904 | .296 |
| I feel there is enough training about how to spot potential signs and symptoms of cancer. | .894 | .355 | .512 |
| I believe I can positively influence my patients’ cancer outcomes | .678 | .712 | .092 |
| I feel there is enough training about how to talk to patients/ customers about cancer in general. | .902 | .313 | .395 |
| I feel I have the knowledge to spot potential signs and symptoms of cancer | .672 | .720 | .442 |
| I feel I have the time to discuss and carry out necessary examinations to spot potential signs and symptoms of cancer | .550 | .414 | .925 |
| Extraction Method: Principal Component Analysis.  Rotation Method: Oblimin with Kaiser Normalization. | | | |

**Appendix 2B.** Factor analysis output for ‘Encouraging patients to spot and respond to potential signs and symptoms of cancer’

| **Structure Matrix** | | | |
| --- | --- | --- | --- |
|  | Component | | |
|  | 1 | 2 | 3 |
| I feel confident helping people make an informed decision about participating in Bowel cancer screening | .853 | -.046 | .450 |
| I would like to take a more active role in helping people make an informed decision about participating in Bowel cancer screening | -.114 | .996 | -.148 |
| I feel I have the knowledge to help people make an informed decision about participating in Bowel cancer screening | .890 | -.068 | .446 |
| I feel I have the time to help people make an informed decision about participating in bowel cancer screening | .476 | -.162 | .984 |
| I feel I have the skills to help people make an informed decision about participating in bowel cancer screening | .887 | -.057 | .498 |
| There is enough training available about how I can counsel people about the benefits and risks of participating in bowel cancer screening | .785 | -.147 | .223 |
| Extraction Method: Principal Component Analysis.  Rotation Method: Oblimin with Kaiser Normalization. | | | |
