# Supplementary figures and images for: Demographic and psychological predictors of community pharmacists’ cancer-related conversations with patients: a cross-sectional analysis and survey study
Source: BMC Health Serv Res. 2022 Feb 28;22:268. doi: 10.1186/s12913-022-07587-1 (PMC8883634; doi:10.1186/s12913-022-07587-1)

**Appendix 3.** NHS Health Research Authority Decision Tool Results.

**
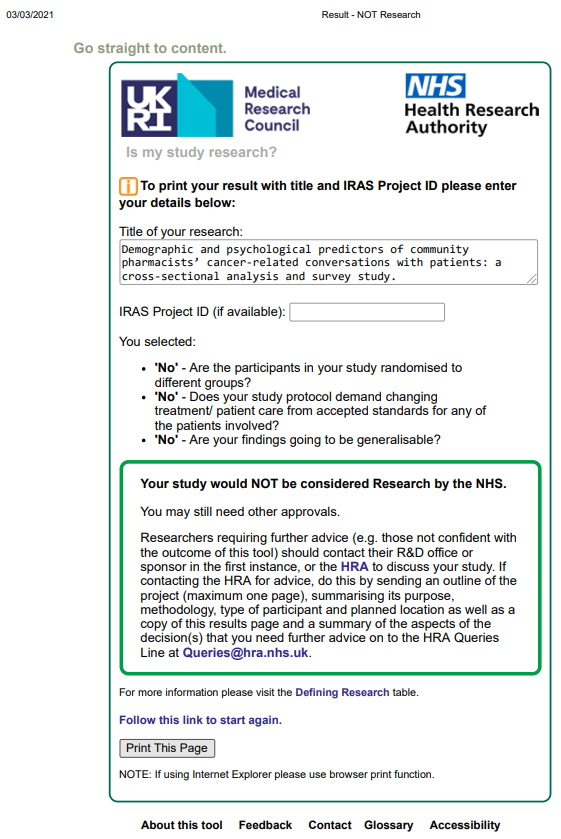
**

Supplement: Supplementary file 3 — Additional file 3. [file 12913_2022_7587_MOESM3_ESM.docx]
