# Supplementary material for: Demographic and psychological predictors of community pharmacists’ cancer-related conversations with patients: a cross-sectional analysis and survey study
Source: BMC Health Serv Res. 2022 Feb 28;22:268. doi: 10.1186/s12913-022-07587-1 (PMC8883634; doi:10.1186/s12913-022-07587-1)
Supplement: Supplementary file 4 — Additional file 4. [file 12913_2022_7587_MOESM4_ESM.docx]

| **Appendix 4.** Ordinal regression analysis results. | | | | |
| --- | --- | --- | --- | --- |
|  | **Appendix 4A.** Predictors of encouraging people to spot and/ or respond to potential signs and symptoms of cancer? | | **Appendix 4B.** Predictors of encouraging people to spot and/ or respond to potential signs and symptoms of cancer? | |
|  | **Expected b (95%CI)** | **P Value** | **Expected b (95%CI)** | **P Value** |
| **Demographic factors** | | | | |
| **Gender** | | | | |
| Male | 0^a^ | **-** | 0^a^ | **-** |
| Female | -1.05 (-1.47, -0.63) | **<0.001** | -0.25 (-0.66, 0.15) | 0.221 |
| **Ethnicity** | | | | |
| White British/Irish/Other | 0^a^ | **-** | 0^a^ | **-** |
| Any other ethnicity | 0.07 (-0.36, 0.50) | 0.761 | 0.42 (-0.002, 0.84) | 0.051 |
| **Region** | | | | |
| England | 0^a^ | **-** | 0^a^ | **-** |
| Devolved nations of the UK: Northern Ireland, Scotland, Wales | 0.16 (-0.40, 0.71) | 0.579 | 0.32 (-0.24, 0.87) | 0.259 |
| **Age** | | | | |
| Years (Continuous) | -0.04 (-0.11, 0.03) | 0.293 | -0.02 (-0.09, 0.05) | 0.611 |
| **Number of years qualified** | | | | |
| Years (Continuous) | 0.07 (-0.004, 0.14) | 0.065 | 0.03 (-0.04, 0.10) | 0.371 |
| **TDF / COM-B Factors** | | | | |
| Capability (4 – 20) | 0.15 (0.07, 0.24) | **<0.001** | **0.34 (0.27, 0.41)** | **<0.001** |
| Opportunity (1 – 5) | 0.23 (-0.002, 0.47) | 0.052 | **0.22 (0.01, 0.42)** | **0.037** |
| Motivation (1 – 5) | 0.47 (0.31, 0.64) | **<0.001** | **0.49 (0.24, 0.74)** | **<0.001** |
|  | R2 = 0.35 | | R2 = 0.41 | |
| * P<0.05  ** P<0.01 *** P<0.001 | | | | |
